# Modulation of Post-Traumatic Immune Response Using the IL-1 Receptor Antagonist Anakinra for Improved Visual Outcomes

**DOI:** 10.1089/neu.2019.6725

**Published:** 2020-05-27

**Authors:** Lucy P. Evans, Addison W. Woll, Shu Wu, Brittany P. Todd, Nicole Hehr, Adam Hedberg-Buenz, Michael G. Anderson, Elizabeth A. Newell, Polly J. Ferguson, Vinit B. Mahajan, Matthew M. Harper, Alexander G. Bassuk

**Affiliations:** ^1^ Department of Pediatrics, University of Iowa, Iowa City, Iowa, USA.; ^2^ Medical Scientist Training Program, University of Iowa, Iowa City, Iowa, USA.; ^3^ Department of Psychiatry, University of Iowa, Iowa City, Iowa, USA.; ^4^ The Iowa City Department of Veterans Affairs Center for the Prevention and Treatment of Visual Loss, Iowa City, Iowa, USA.; ^5^ Department of Molecular Physiology and Biophysics, and University of Iowa, Iowa City, Iowa, USA.; ^6^ Department of Ophthalmology and Visual Sciences, University of Iowa, Iowa City, Iowa, USA.; ^7^ Omics Laboratory, Byers Eye Institute, Department of Ophthalmology, Stanford University School of Medicine, Palo Alto, California, USA.; ^8^ Veterans Affairs Palo Alto Health Care System, Palo Alto, California, USA.

**Keywords:** anakinra, blast, IL-1, retina, visual function

## Abstract

The purpose of this study was to characterize acute changes in inflammatory pathways in the mouse eye after blast-mediated traumatic brain injury (bTBI) and to determine whether modulation of these pathways could protect the structure and function of retinal ganglion cells (RGC). The bTBI was induced in C57BL/6J male mice by exposure to three 20 psi blast waves directed toward the head with the body shielded, with an inter-blast interval of one hour. Acute cytokine expression in retinal tissue was measured through reverse transcription-quantitative polymerase chain reaction (RT-qPCR) four hours post-blast. Increased retinal expression of *interleukin* (*lL)-1β, IL-1α, IL-6,* and *tumor necrosis factor (TNF)α* was observed in bTBI mice exposed to blast when compared with shams, which was associated with activation of microglia and macroglia reactivity, assessed via immunohistochemistry with ionized calcium binding adaptor molecule 1 and glial fibrillary acidic protein, respectively, one week post-blast. Blockade of the IL-1 pathway was accomplished using anakinra, an IL-1RI antagonist, administered intra-peritoneally for one week before injury and continuing for three weeks post-injury. Retinal function and RGC layer thickness were evaluated four weeks post-injury using pattern electroretinogram (PERG) and optical coherence tomography (OCT), respectively. After bTBI, anakinra treatment resulted in a preservation of RGC function and RGC structure when compared with saline treated bTBI mice. Optic nerve integrity analysis demonstrated a trend of decreased damage suggesting that IL-1 blockade also prevents axonal damage after blast. Blast exposure results in increased retinal inflammation including upregulation of pro-inflammatory cytokines and activation of resident microglia and macroglia. This may explain partially the RGC loss we observed in this model, as blockade of the acute inflammatory response after injury with the IL-1R1 antagonist anakinra resulted in preservation of RGC function and RGC layer thickness.

## Introduction

Traumatic brain injury (TBI) is a leading cause of death and disability worldwide, which results in enormous social and economic costs. Because of the use of improvised explosive devices in 21st century military conflicts, the number of blast-related injuries causing TBI (bTBI) has increased dramatically in both military personnel and civilians, while blast-related deaths have decreased because of enhanced protective equipment.^1,2^ Unfortunately, there are no effective pharmacological therapies to prevent neuronal loss after blast, and treatment is limited to supportive care.

The retina is a central nervous system (CNS) tissue vulnerable to injuries affecting the brain.^3^ This is true particularly in the setting of bTBI because the eye is also exposed to the primary blast wave. Many military personnel and civilians who have bTBI also report symptoms of visual dysfunction with retinal pathology, which can present either acutely or chronically after the initial injury.^1,4^ Although patients with bTBI report a wide range of visual disturbances, little is known about the molecular mechanisms driving visual dysfunction. Murine bTBI models display many of the long-term visual deficits observed in patients, particularly retinal ganglion cell (RGC) dysfunction and subsequent cell death.^5–14^

After a TBI, secondary signaling cascades occur in the brain, including robust neuroinflammation,^15^ which can trigger progressive neuronal dysfunction, neuronal death, and tissue destruction that exacerbates the initial injury. A therapeutic window exists in the hours to days after initial trauma when inflammatory pathways are initiated and can be blocked to halt progressive tissue injury. Components implicated in this neuroinflammation include rapid upregulation of the interleukin (IL)-1 cytokine family, including IL-1α and IL-1β.^16–20^ The IL-1 cytokines can be produced via activated resident macroglia (astrocytes and Müller glia) and microglia, or through infiltration of systemic immune cells into the CNS.^21^

Signaling through the primary receptor for IL-1, IL-1RI, results in the activation of the transcription factor nuclear factor-κB (NF-κB) and the stress-related mitogen-activated protein kinases (MAPKs), ultimately resulting in the upregulation of inflammation-associated genes including, but not limited to, IL-8, IL-6, TNFα, PGE2, and nitric oxide.^22^ The IL-1R1 is present on many retinal cell types, such as microglia, macroglia, endothelial cells, neurons (including RGCs), and invading peripheral immune cells. Increased signaling through these cell types may contribute further to neuronal injury.^21,23,24^

Several pre-clinical studies regarding the brain have begun to test blockade of IL-1β after TBI, including use of anti-IL-1β antibody or blockade of IL-1RI. Encouraging results showed both histological and functional improvement with decreased tissue loss and attenuated cognitive deficits.^25–28^ Anakinra, a recombinant human IL-1 receptor antagonist (rhIL-1Ra) that mimics the action of the natural antagonist IL-1Ra, is also being studied as a potential therapy for patients with TBI. In a phase-2 trial of adults with severe TBI, anakinra had a good safety profile and brain penetration when administered peripherally.^29^

Within the retina, microglial activation is believed to contribute to retinal injury after trauma by reacting to and propagating damaging secondary inflammation. The presence of activated/reactive microglia within the retina after blast injury specifically has been reported,^30,31^ in addition to being seen in other types of retinal injury such as in weight drop TBI models^32^ and optic nerve injury models.^33,34^ Microglia can exist along a continuous scale between three distinct states: (1) an inactivated state with a ramified morphology and processes constantly monitoring the environment for injury signals; (2) an activated proinflammatory and cytotoxic M1-state; and (3) an activated anti-inflammatory and reparative M2-state.^35^

Murine bTBI studies have shown that shifting microglia away from the proinflammatory M1 state to the protective M2 state can help prevent secondary injury after blast.^11–13^ This suggests that microglial polarization toward the harmful M1 state plays a role in bTBI retinal pathogenesis and that retinal inflammation is a viable therapeutic target.

The subsequent questions that remain are: which inflammatory molecules play a role in the damaging neuroinflammation specifically within the retina and is it possible to pharmacologically target these inflammatory molecules in bTBI patients to prevent long-term visual dysfunction? While the IL-1 pathway is involved in secondary neuroinflammation in the brain, investigating this pathway within the eye is a logical first step in understanding the retinal response after blast injury. In this study, we developed a model of repeated blast injury that results in visual impairment in the form of decreased RGC signaling and RGC layer thickness, consistent with what has been seen with single blast injury in mice.^6,36^

We then identified molecular mediators of inflammation in the retina, which have long been implicated in inflammatory damage in the brain after TBI, and confirmed that retinal injury could be detected non-invasively using clinical testing modalities used in humans. Finally, we rescued this damage using a pharmacological agent blocking the IL-1 receptor (IL-1RI), an effect that could be monitored by retinal examination. Taken together, this points to a diagnostic and therapeutic strategy for blast-induced retinal injury.

## Methods

### Animals

All animal studies were conducted in accordance with the ARVO Statement for the Use of Animals in Ophthalmic and Vision Research and were approved by the University of Iowa and Iowa City Veterans Affairs Institutional Animal Care and Use committee. Studies were conducted on male C57BL/6J mice purchased from The Jackson Laboratory (Bar Harbor, ME) aged 2–4 months, with an average weight of 26.4 ± 1.3 g. A total of 125 mice were used for this study, and all were assigned randomly to injury conditions and treatment groups. Some mice were utilized to validate the retinal response to repetitive bTBI, while others were used to test the effects of anakinra (Supplementary Table S1). Mice were housed under a 12-h light-dark cycle with *ad libitum* access to food and water.

### Blast injury induction

An enclosed blast chamber was used, one half of which was pressurized, as described previously.^36^ A plastic Mylar membrane (Mylar A, 0.00142 gauge; Vountry Plastics, Ames, IA) was placed over a 13-cm opening that separates the sides of the chamber. The unpressurized side of the tank contained a padded polyvinyl chloride (PVC) protective restraint in which to place an anesthetized mouse 30 cm from the Mylar membrane. Compressed air was pumped into the pressurized side of the chamber until the membrane ruptured at 20 ± 0.2 psi (137.8 ± 1.3 kPa, mean ± standard error of the mean [SEM]), creating a blast wave. Because many veterans are exposed to multiple blast exposures, we administered three injuries to each mouse to mimic human injuries.^10,37–39^

**FIG. 1.**
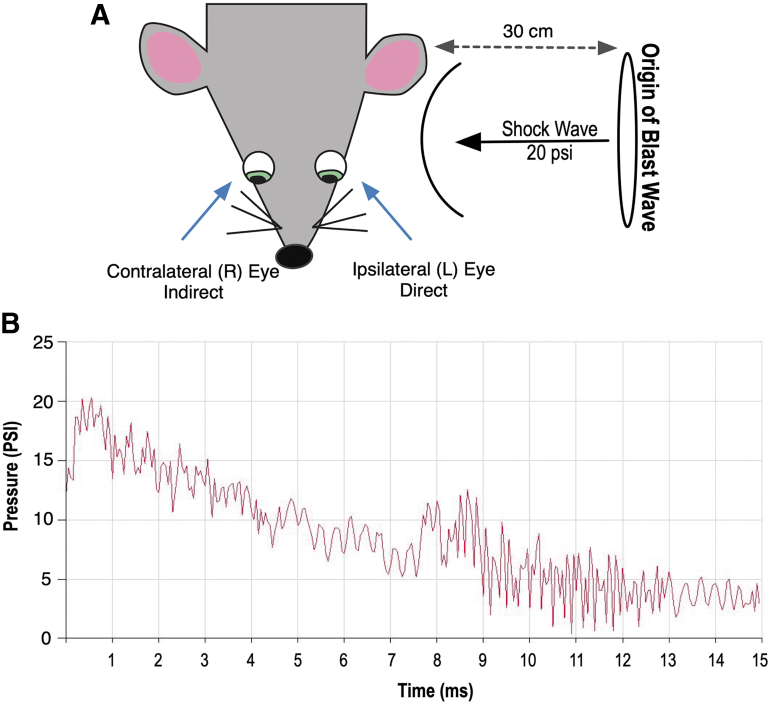
Schematic representation of blast exposure. Exposure to blast wave pressure was conducted as described previously.^23^ Animals were placed lateral to the shock tube axis, 30 cm from the origin of the blast wave (Mylar membrane), with the left (L) side of the head (ipsilateral eye) facing the blast wave. (**A**) Control mice were restrained the same way, but were not exposed to the blast wave. (**B**) A representative tracing of a 20 ± 0.2 psi (137.8 ± 1.3 kPa, mean ± SEM) blast wave.

Mice were oriented within the chamber with the left side of the head positioned toward the blast wave (direct exposure) and the right side facing away from the direction of the blast wave (indirect exposure) and then exposed to three blast injuries, each 1 h apart (Fig. 1). The mouse's body was shielded via the PVC restraint to limit blast wave pressure exposure primarily to the head; the head was allowed to move freely and was not in a fixed position. All analysis was conducted on the left (ipsilateral) side that was exposed directly to the blast wave, because we cannot discount potential interaction of the contralateral eye with the padded holder, or potential confounding, rebounding blast waves from the surface of the animal holder.

Mice were anesthetized with a combination of ketamine (30 mg/kg, intraperitoneal [IP]) and xylazine (5 mg/kg, IP) before each blast or sham-blast and were placed on a heating pad immediately after blast injury to prevent hypothermia and facilitate recovery from general anesthesia. Xylazine anesthesia was reversed with yohimbine chloride (0.001 mg/g, IP) to aid in recovery from anesthesia. Control mice underwent an identical process in all respects except that they did not receive a blast exposure when placed in the chamber. Both blasted and sham mice were given an IP injection (0.1 mL/20g body weight) of buprenorphine (0.003 mg/mL) immediately after the blast or sham-blast, respectively.

### Regional brain dissection

Brains from sham and blast mice were isolated and regionally dissected to generally separate major portions of the brain. The cerebellum (CB) and brainstem (BS; includes the midbrain and hindbrain) were isolated. Standard dissection of the hippocampus (HIPP) was then performed,^40^ followed by separation of the cortex (CTX). What remained was the extra forebrain (XFB) tissue block containing striatum, thalamus, hypothalamus, and visual white matter tracts. The samples harvested were used to determine the effects of blast injury on cytokine expression in the brain via real time-quantitative polymerase chain reaction (RT-qPCR, described below).

### Ribonucleic acid (RNA) isolation, complementary deoxyribonucleic acid (cDNA) preparation, and RT-qPCR

Total RNA was extracted from retinas at 4 and 24 h post-blast. Retinas were lysed with a homogenizing pestle and filtered through QiaShredder columns (Qiagen, Chatsworth, MD). The RNA was then extracted with an RNeasy Mini Kit (Qiagen) according to the manufacturer's instructions. Brains from sham and blast mice were isolated and regionally dissected as described above. Total RNA was extracted from brain regions using TRIzol (Invitrogen, Carlsbad, CA) as per the manufacturer's instructions.

For cDNA preparation of retinas and brain regions, respectively, 1000 ng or 2000 ng of total RNA was reversed transcribed using random hexamers and SuperScript III Reverse Transcriptase (Invitrogen). Amplified cDNAs were diluted 1:10 and 1:15 for retina and brain samples, respectively, in ultrapure water and subjected to RT-PCR using an Applied Biosystems Model 7900HT with TaqMan Universal PCR Mastermix (Applied Biosystems) with the following probes: *IL-1β* (Mm00434228_m1), *IL-1α* (Mm00439620_m1), *IL-6* (Mm00446190_m1), *tumor necrosis factor* (*TNF)-α* (Mm00443258_m1), and *GAPDH* (4308313). Biological samples were run in triplicate. Relative messenger RNA (mRNA) levels of target genes were normalized to the endogenous control, *GAPDH*, using the comparative cycle threshold method.

Results were expressed as fold difference from sham controls. Column statistics were run to determine whether values were distributed normally. For those with normal distribution, a Student *t* test was used; otherwise, a Mann-Whitney *U* test was utilized.

### Immunohistochemistry (IHC)

For sample preparation for IHC experiments, blast-exposed and sham mice were anesthetized deeply with ketamine 200 mg/kg and xylazine 20 mg/kg and perfused transcardially with 0.01 mol/L phosphate-buffered saline (PBS) followed by 4% paraformaldehyde in 0.01 mol/L PBS. Ipsilateral globes were post-fixed in 4% paraformaldehyde for 1 h at room temperature.

Retinal cross sections were prepared for examining glial fibrillary acidic protein (GFAP) immunostaining. To this end, the lens and cornea were removed and posterior eyecups were subjected to a sucrose gradient (10%, 20%, and 30% in 0.01 mol/L PBS), embedded in optimal cutting temperature (OCT) compound, and frozen using dry ice. Sagittal sections (8 μm thick) of the ipsilateral retina were collected onto superfrost plus glass slides. The OCT-embedded sections were hydrated for 15 min with tris-buffered saline with 0.05% Tween 20, followed by 45 min of permeabilization with 0.3% triton in PBS. Sections were blocked for 1 h with 3% goat serum/0.1% Triton X-100 (cat. no. T8532; Sigma-Aldrich, St. Louis, MO) in PBS.

Primary antibodies against GFAP (1:400; cat. no. Mab360; Millipore, Burlington, MA) were diluted in blocking solution and costained with fluoresceinated Griffonia simplicifolia type 1 Isolectin B4 (Alexa Fluor 594 conjugated, 1:100, cat. no. 121413; Life Technologies, Grand Island, NY) in 1 mM CaCl2 in PBS overnight at 4°C. Tissues were washed with PBS (3** ×** 5 min) and incubated with corresponding secondary antibody GAM647 (1:300; cat. no. A-32728; Thermo Fisher Scientific) with Isolectin B4 (1:100) in 3% goat serum/0.1% triton in PBS for 1 h at room temperature. Samples were washed with PBS (3** ×** 5 min), incubated with DAPI (4 μg/mL; cat. no. D9564; Sigma-Aldrich) for 10 min at room temperature, and washed with PBS (3** ×** 5 min).

For ionized calcium binding adaptor molecule 1 (IBA-1-) and GFAP-stained whole-mounted retinas, retinal dissection was followed by permeabilization with 0.3% Triton X-100 in PBS for 1 h at room temperature and antigen retrieval treatment (10 mM sodium citrate, pH = 6 with 0.05% Tween 20) at 90°C for 10 min. Retinas were blocked overnight with 3% goat serum/0.1% triton in PBS at 4°C, and then incubated for 1.5 h with Background Buster (cat. no. NB306; Innovex Biosciences Inc., Richmond, CA).

Retinas were incubated with GFAP antibodies as described above and with the primary antibody for IBA-1 (1:500; cat. no. 019-1941; Wako Chemicals USA, Inc., Richmond, VA) overnight at 4°C, washed with PBS (3** ×** 5 min), and incubated with the corresponding secondary antibody GAR488 (1:300; cat. no. A11070; Thermo Fisher Scientific, Waltham, MA) for 1 h at room temperature. All slides were mounted with ProLong Gold Antifade Mountant (cat. no. P36934; Thermo Fisher Scientific) followed by imaging with confocal microscopy (SP8 confocal, Leica Microsystems, Wetzlar, Germany).

### Quantification of IBA-1 and GFAP IHC staining

**FIG. 2.**
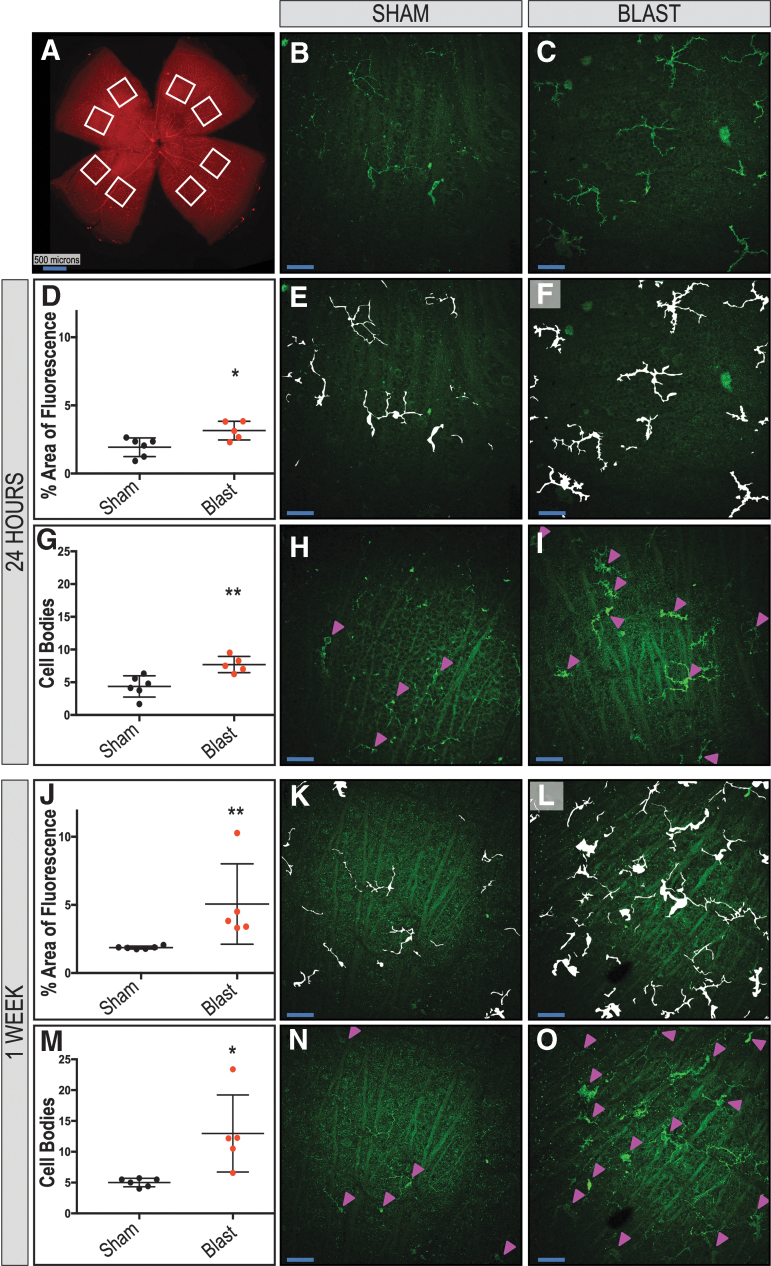
Increased immunoreactivity and distribution of IBA-1-positive microglia are found in retinas exposed to blast. Eight regions were sampled per ipsilateral retina (**A**). Quantification shows an increase in total fluorescence area (**D, J**) and cell body counts (**G, M**) after blast in the retinal nerve-fiber layer (RNFL) and retinal ganglion-cell layer of whole-mounted retinas at 24 h and one week, respectively. Representative images (**B, C**) and mask overlays of area are quantified (**E, H, K, M** and **F, I, L, O**) for sham and blast-injured mice, respectively. Arrowheads indicate cell bodies (**H, I, N, O**). Student *t* test or Mann-Whitney *U* test are based on distribution of data. *p < 0.05; **p < 0.01. Data are expressed as means ± SEM. *n* = 5–6 mice per group. X25, original magnification; *en face* view with the RNFL facing up. Scale bar: 50 μm unless otherwise noted.

Whole-mounted retinas were stained as described above. Two non-overlapping images were collected for each of four retinal petals, yielding a total of eight images per retina, and were taken midway between the outer edge of the petal and the optic nerve head (Fig. 2A). Images were taken using a X25 water-immersion objective with consistent microscope settings to eliminate variation from one sample to the next. Quantitative analysis of fluorescent area for IBA-1+ staining was performed using ImageJ Imaging Software (National Institutes of Health, Bethesda, MD),^41^ and microglial cell counts were completed manually by an observer masked to the origin of the samples. As a reference, the masked observer utilized Z-stack images of the areas below and above the image being quantified to assist in determining what was an IBA-1+ microglia cell body that colocalized with the nuclear marker, DAPI.

The process was performed systematically for all of the images. Overlay masks were hand drawn, and any clear artifacts or debris was removed. Values from the eight images were averaged, producing one value per retina.

**FIG. 3.**
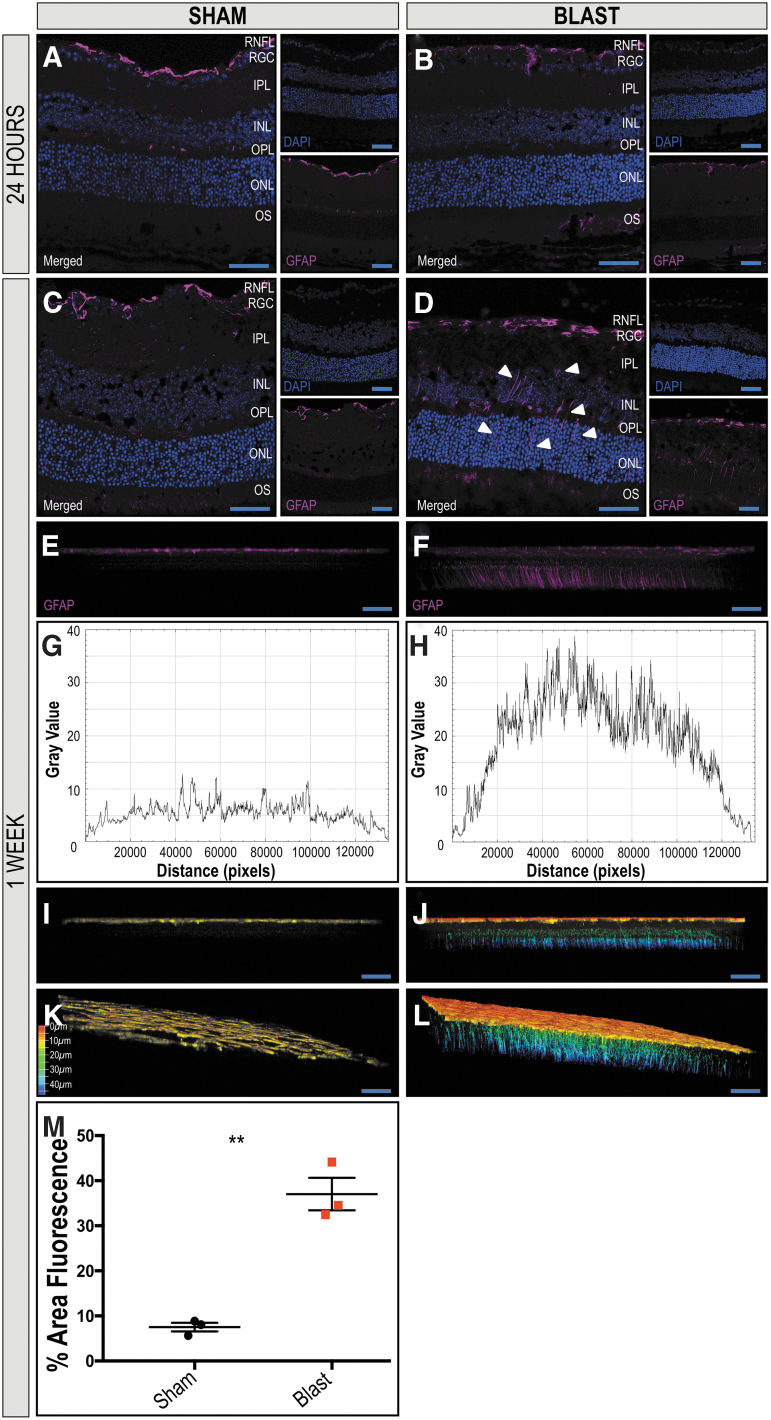
Extended distribution of glial fibrillary acidic protein (GFAP) in Müller glia in retinas ipsilateral to blast exposure suggests increased damage. The GFAP localizes to the retinal nerve fiber layer (RNFL) of retinas of sham and blast mice 24 h after injury (**A, B,** respectively). One week after blast exposure, the distribution of GFAP immunoreactivity remains in the RNFL of sham mice (**C**), but increases in Müller glia spanning the retina from the RNFL to the outer nuclear layer (ONL) in blast mice (**D**) in retinal cross sections. A two-dimensional side facing view of a reconstructed z-stack acquired repeatedly in 0.5 μm confocal sections along the z-axis of retinal whole mounts demonstrates GFAP reactivity limited to the RNFL of sham mice (**E**) and spanning throughout the retina in blast mice (**F**). A plot-profile displaying pixel intensity of the GFAP staining in **E** and **F** demonstrates a quantitative increase in intensity in the blast retina **(H**) when compared with sham (**G**). Figures **I–L** are depth coded images demonstrate GFAP+ extension into deeper retinal layers of blast mice (**J, L**) when compared with sham (**I, K**). Color scale bar corresponds to the z-axis depth of GFAP positive cells as the distance from the vitreous, coded from red at 0 μm and blue at 50 μm. Quantification (**M)** demonstrates a significant increase in total fluorescent area in the retinas from blast-injured mice when compared with sham-injured mice. Significance was determined via Student *t* test (**p = 0.0014). Data expressed as means ± SEM. *n* = 3 per condition. X25, original magnification; scale bar: 50 μm. RGC, retinal ganglion-cell layer; IPL, inner plexiform layer; INL, inner nuclear layer; OPL, outer plexiform layer; OS, photoreceptor outer segments.

**FIG. 4.**
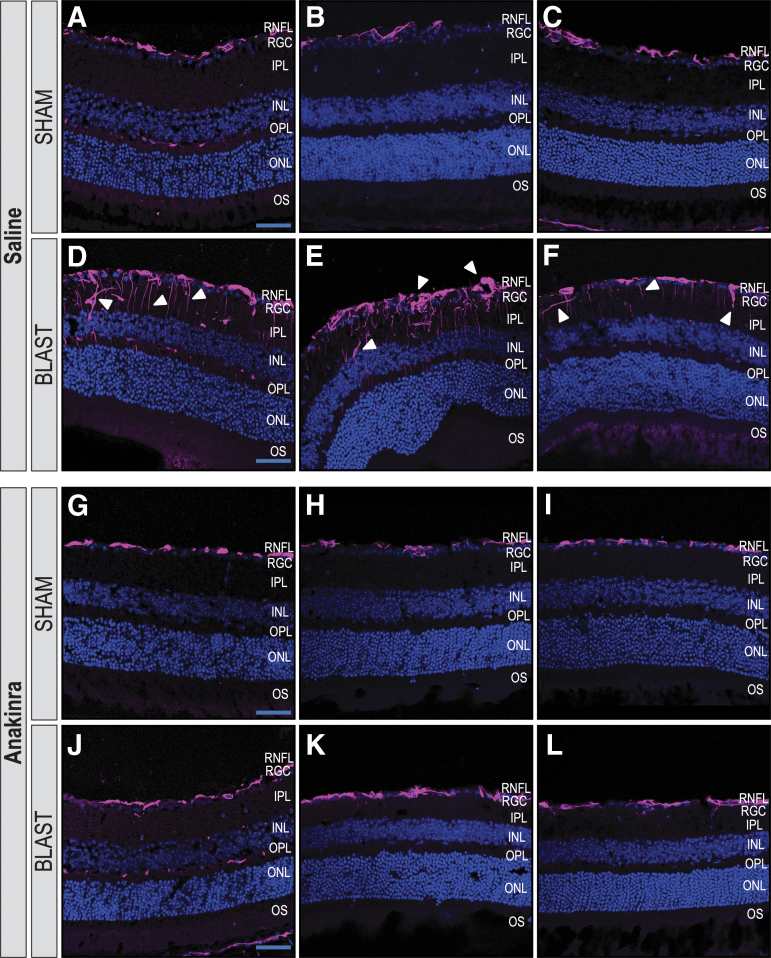
Anakinra treatment prevents Müller glia activation in the retina after blast. Distribution of glial fibrillary acidic protein (GFAP) in retinas ipsilateral to blast exposure are seen four weeks after injury. Images of areas with the highest GFAP reactivity are shown in retinal cross sections of three individual mice per group. The GFAP localizes to the retinal nerve fiber layer (RNFL) of retinas of sham-saline (**A–C**) and sham-anakinra (**D–F**) mice four weeks after blast injury. Retinas from blast-saline mice demonstrate increased GFAP immunoreactivity in Müller glia spanning the retina from the RNFL into deeper layers (**G–I**). Blast-anakinra mice, however, show decreased GFAP immunoreactivity overall (**J–L**) when compared with the injury group only given saline. *n* = 3 mice per group. RGC, retinal ganglion-cell layer; IPL, inner plexiform layer; INL, inner nuclear layer; OPL, outer plexiform layer; ONL, outer nuclear layer; OS, photoreceptor outer segments.

**FIG. 5.**
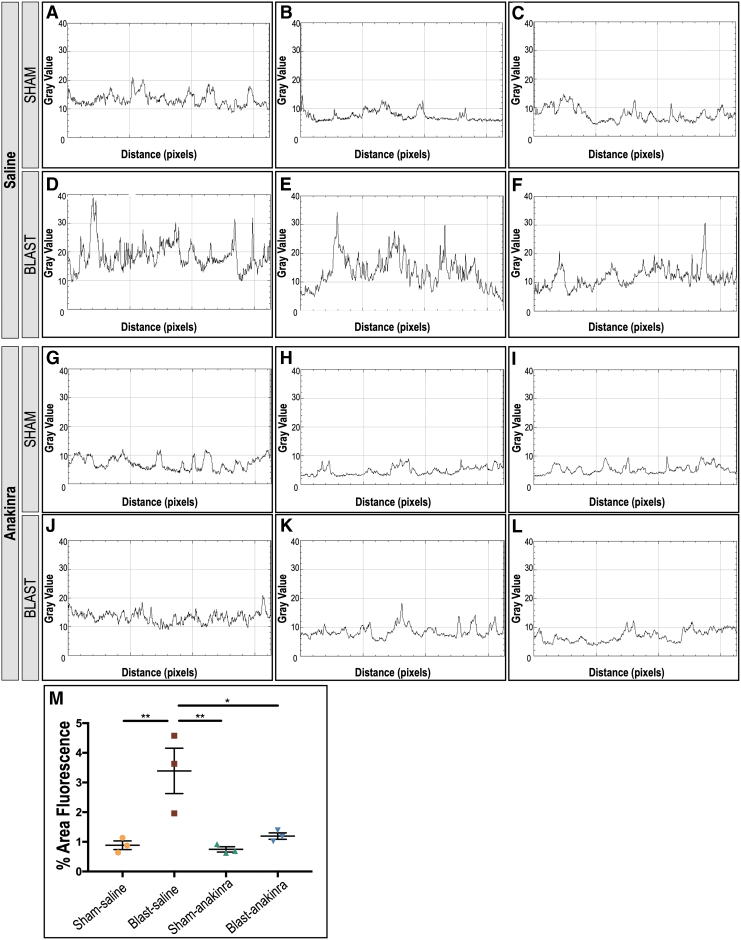
Quantification of glial fibrillary acidic protein (GFAP) suggests anakinra treatment prevents Müller glia activation in the retina after blast. Plot-profiles display pixel intensity of the GFAP staining in retinal cross section images from Figure 4. Blast-saline (**D–F**) images demonstrate increased GFAP intensity compared with sham-saline (**A–C**), sham-anakinra (**G–I**), and blast-anakinra (**J–L**). Quantification (**M)** demonstrates a significant increase in the total fluorescent area in the retinal sections from blast-saline mice when compared with all other groups. No other significant differences were found. Significance was determined by comparing means of all groups using one-way analysis of variance with the Dunnett post-test (*p < 0.05, **p < 0.01). Data expressed as means ± SEM. *n* = 3 per condition.

To compare GFAP expression between sham and blast mice at one week post-injury, ImageJ was used to generate plot profiles reflective of pixel intensity and to quantify the percent fluorescent area. A two-dimensional side facing view of a reconstructed Z-stack acquired repeatedly in 0.5 μm confocal sections along the z-axis of retinal whole mounts was used, and total area selected was consistent for all images (Fig. 3; *n* = 3 per condition). For GFAP analysis after anakinra treatment, ImageJ was also used to generate plot profiles reflective of pixel intensity of retinal cross sections and quantitative analysis of the fluorescent area (Fig. 4, 5; *n* = 3 per condition).

### Treatment with anakinra

**FIG. 6.**
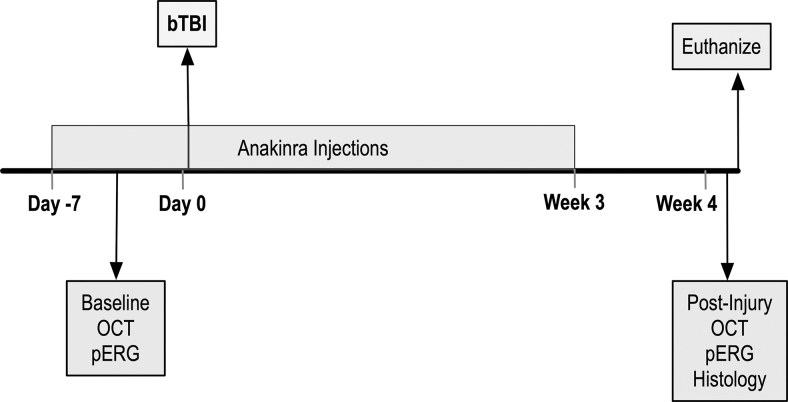
Experimental design of anakinra treatment. Mice were given daily intraperitoneal injections of either saline or anakinra beginning one week before injury and continuing three weeks after. Optical coherence tomography (OCT) and pattern electroretinogram (PERG) were conducted before injury as a pre-blast baseline and four weeks after, followed by euthanasia and tissue collection for histological analysis of retinal and optic nerve tissue.

Blast and sham mice were assigned randomly to receive IP injections once daily with a dose of 100 mg/kg or an equal volume of sterile 0.9% saline. Anakinra (67 mg/mL; Sobi) was diluted in sterile 0.9% saline. Treatment was initiated one week before blast injury and continued for three weeks post-injury (Fig. 6). Researchers were blinded to the treatments for the entirety of the study including drug administration, data acquisition, and data analysis.

### Pattern-evoked electroretinography (PERG)

The PERG was used to objectively measure the function of RGCs by recording the amplitude of the PERG waveform after TBI. Mice were anesthetized with a combination of ketamine (0.05–0.06 mg/g, IP), xylazine (0.008–0.01 mg/g, IP), and acepromazine (0.004–0.005 mg/g, IP), and then placed on a heated recording table to maintain body temperature. Neutral position PERG responses were evoked using alternating, reversing, and black and white vertical stimuli delivered on a monitor with a Roland Consult ERG system (Roland Consult, Brandenburg, Germany).

To record the PERG response, commercially available stainless steel subdermal electrodes (Ambu, Ballerup, Denmark) were placed in the snout. A reference needle electrode was placed medially at the base of the head, and a ground electrode was placed at the base of the tail to complete the circuit. Each animal was placed at the same fixed position in front of the monitor to prevent recording variability because of animal placement.

Stimuli (18 degree radius visual angle subtended on full field pattern, two reversals per second, 372 averaged signals with cutoff filter frequencies of 1 to 30 Hz, 98% contrast, 80 cd/m^2^ average monitor illumination intensity (Jorvec, Miami, FL) were delivered under mesopic conditions without dark adaptation to exclude the possible effect of direct photoreceptor-derived evoked responses. A diffuser placed over the pattern on the monitor also did not elicit a measurable evoked potential, further ensuring that the electrical responses were elicited from RGCs.

The PERG response was evaluated by measuring the amplitude (peak to trough) of the waveform, as we have described previously.^8,36^ The PERG response was recorded at pre-blast baseline, and four weeks after blast injury. Significance was determined comparing means of all groups using Kruskal-Wallis test with the Dunn post-test. Exclusions were made for mice that were breathing too heavily or if their temperature dropped during acquisition, which was monitored constantly throughout the experiment. Changes in each of these parameters can cause interference in signal acquisition. Exclusion criteria were decided before data acquisition for an individual mouse.

### Spectral-domain optical coherence tomography (SD-OCT)

The SD-OCT analysis was performed at pre-blast baseline and four weeks post-injury using a Spectralis SD-OCT (Heidelberg Engineering, Vista, CA) imaging system and a 25 dipoter (D) lens for mouse ocular imaging (Heidelberg Engineering). Mice were anesthetized with a combination of ketamine (0.03 mg/g, IP) and xylazine (0.005 mg/g, IP) and placed on a heating pad to maintain body temperature. Pupils were dilated using a 1% tropicamide solution, and the cornea was moisturized with saline. Volume scans (49 line dense array) positioned directly over the optic nerve head were performed to quantify the RGC complex thickness, which includes RGC bodies, axons, and dendrites.

Scans approximately 100 um from the edge of the optic nerve head were analyzed by a masked observer, excluding blood vessels from the RGC complex thickness calculation. Statistical significance was determined by one-way ANOVA with the Dunnett post-test for multiple comparisons.

### Histology and microscopy of optic nerves

Blast-exposed and sham-blast mice were anesthetized deeply with carbon dioxide, lightly perfused with normal saline followed by 4% paraformaldehyde and euthanized by decapitation. Optic nerves were collected and processed as described previously.^42^ In brief, optic nerves were dissected from heads and drop fixed in half-strength Karnovsky fixative (2% paraformaldehyde, 2.5% glutaraldehyde in 0.1 M sodium cacodylate) at 4°C for 16 h. Nerves were rinsed in 0.1 M Na cacodylate buffer, post-fixed with 1% osmium tetroxide, dehydrated in graded acetone (30–100%), infiltrated in graded resin (33%, 66%, and 100%; Eponate-12; Ted Pella, Redding, CA) diluted in propylene oxide, embedded in fresh 100% resin, and then polymerized in a 65°C oven.

Semithin (1-μm) cross sections were cut, transferred to glass slides, stained with 1% paraphenylenediamine (PPD), and mounted to glass slides (Permount, Fisher Scientific, Pittsburgh, PA). Light micrograph images were obtained with an Olympus BX-52 microscope (Cedar Valley, PA) at total magnifications of 100X and 1000X.

### Grading of optic nerve damage

Assessing levels of damage in optic nerves was performed using a three-level grading scale (1-none to mild damage, 2-moderate, or 3-severe). This method has been described previously (grading scale, grading criteria, estimated axon counts for each damage grade) and validated extensively.^43–47^ Microscopy slides containing mounted optic nerve cross sections stained with PPD were assessed by two independent investigators who were blinded to both the identity of the nerves and the scores given by the other investigator. Investigators assigned the same score for 91% of the nerves. In the cases of damage grade disagreement, a third investigator, blinded to both the identity of nerves and scores given by the first two investigators, assigned a third damage grade, and the most common grade (among all three investigators) was used in the final grading for each nerve specimen. Representative light micrographs were obtained from slides using a light microscope (BX-52, Olympus) at total magnifications of 100X and 1000X.

### Experimental design and statistical analysis

Supplementary Table S1 provides information concerning all mice utilized in this study. The experimental design of the anakinra study is depicted in Figure 6. Column statistics were run to determine whether values were distributed normally for all quantitative experiments. For the RT-qPCR data and quantification of immunohistochemistry at 4 h, 24 h, and one week, a Student *t* test was used if data were distributed normally; otherwise, a Mann-Whitney *U* test was utilized. Statistical significance for the PERG and OCT analysis was determined by Kruskal-Wallis test with a Dunn post-test and by one-way ANOVA followed by the Dunnett post-test for multiple comparisons, respectively, determined by the distribution of the data. Statistical significance for the IBA-1 quantification of drug treated mice was determined by a one-way ANOVA. A value of *p* < 0.05 was considered significant. All the statistical data were presented as mean ± SEM and were performed using GraphPad Prism 7 for Macintosh (La Jolla, CA).

## Results

### Repeated bTBI causes increased expression of inflammatory cytokines in the retina and brain

**FIG. 7.**
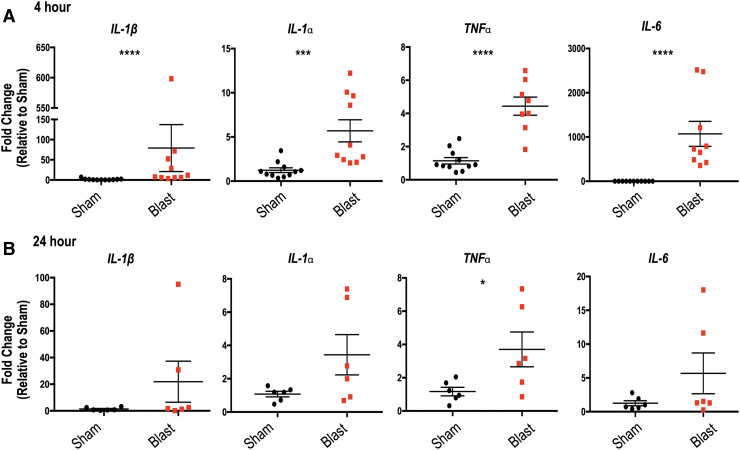
Retinal expression of inflammatory markers is increased 4 h post-repeated blast traumatic brain injury (bTBI). Quantitative polymerase chain reaction measuring mRNA levels relative to glyceraldehyde-3-phosphate dehydrogenase *(GAPDH)* at (**A**) 4 h and (**B**) 24 h post-bTBI. Increased expression of *interleukin* (*IL)-1β*, *IL-1α*, *tumor necrosis factor* (*TNF)α*, and *IL-6* in the ipsilateral retinas of blast-injured mice was seen when normalized to sham values at 4 h after injury. At 24 h, only *TNFα* is increased in the blast retinas. Student *t* test or Mann-Whitney *U* test based on distribution of data. *p < 0.05; ***p < 0.001; ****p < 0.0001. Data expressed as means ± standard error of the mean. *n* = 8–11 and six mice per group at 4 h and 24 h, respectively.

Because robust neuroinflammation occurs in the brain after several types of TBI,^15^ we first examined the mRNA levels of classic proinflammatory cytokines in the retina and brain after bTBI. At 4 h post-repeated bTBI, there was an acute increase in retinal inflammatory cytokine mRNA when normalized to sham values, with increased expression of *IL-1β* (*p* < 0.0001), *IL-1α* (*p* = 0.003), *TNFα* (*p* < 0.0001), and *IL-6* (*p* < 0.0001) in the ipsilateral retinas of blast-injured mice (Fig. 7A). At 24 h post-bTBI, inflammation within the retina had decreased, with only a significant increase in *TNFα* (Fig. 7B; *p* = 0.0405).

**FIG. 8.**
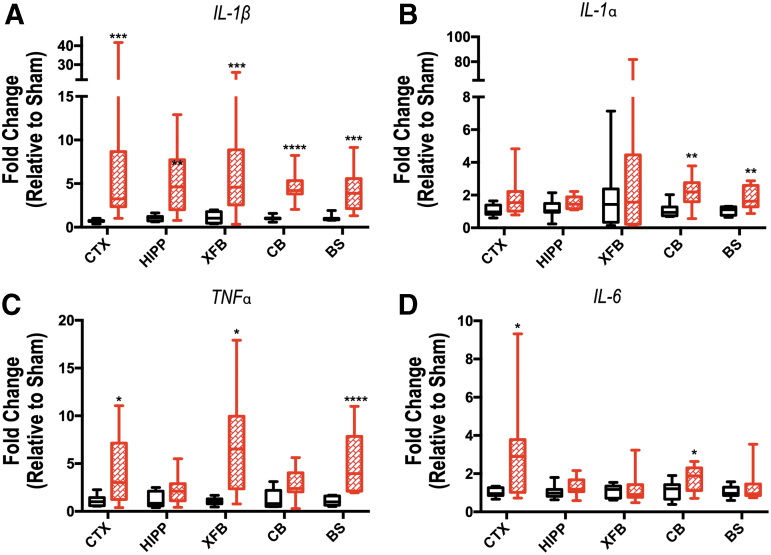
Inflammatory cytokine expression in brain tissue demonstrates regional changes 4 h after repeated blast traumatic brain injury. Expression of *interleukin* (*IL)-1β* (**A**), *IL-1α* (**B**), *tumor necrosis factor* (*TNF)α* (**C**), and *IL-6* (**D**) was evaluated by quantitative polymerase chain reaction in the ipsilateral cortex (CTX), hippocampus (HIPP), extra forebrain (XFB), cerebellum (CB), and brainstem (BS). The mRNA levels are relative to the housekeeping gene *GAPDH* (glyceraldehyde-3-phosphate dehydrogenase). Data are expressed as fold change in gene expression relative to sham and are presented as box and whiskers plots; the box extends from the 25th to the 75th percentiles, the line represents the median, and the whiskers extend from smallest to largest value. Student *t* test or Mann-Whitney *U* test based on distribution of data. *p < 0.05; **p < 0.01; ***p < 0.001; ****p < 0.0001. *n* = 7–8 mice per group.

Increases in *IL-1β*, *IL-1α*, *TNFα*, and *IL-6* were seen in brain tissue of mice exposed to blast injury in a region-dependent manner 4 h after injury. *IL-1β* was increased in all regions analyzed: CTX, HIPP, XFB, CB, and BS samples of bTBI mice (*p* < 0.0002, *p* = 0.0085, *p* = 0.0046, *p* < 0.0001, *p* = 0.0002, respectively; Fig. 8A). *IL-1α* was increased in CB and BS (*p* = 0.0046, *p* = 0.0085, respectively; Fig. 8B), *TNFα* was increased in CTX, XFB, and BS (*p* = 0.0385, *p* = 0.0103, *p* < 0.0001, respectively; Fig. 8C), and *IL-6* was increased in CTX and CB (*p* = 0.0343 and *p* = 0.0489, respectively; Fig. 8D) of blast-injured samples. While inflammatory mRNA expression varied between regions and also between individual samples, these increases are indicative of acute inflammatory changes overall in the brain in addition to those seen in the retina.

### Repeated bTBI induces cellular activation in the retina because of injury

The mammalian retina contains three types of glial cells; microglia are the resident phagocytes of the retina along with two forms of macroglia cells, astrocytes and Müller (radial glial) cells, which carry out neuronal support functions in the retina.^48,49^ A TBI triggers rapid activation of resident microglia and macroglia, all of which can be activated by and continue to produce inflammatory cytokines.^21^ The prevalence and morphology of retinal microglia in the retinal nerve fiber layer (RNFL) and RGC layer in response to blast injury was assessed at 4 h, 24 h, and one week after injury using the marker IBA-1 on retinal whole mounts.

**FIG. 9.**
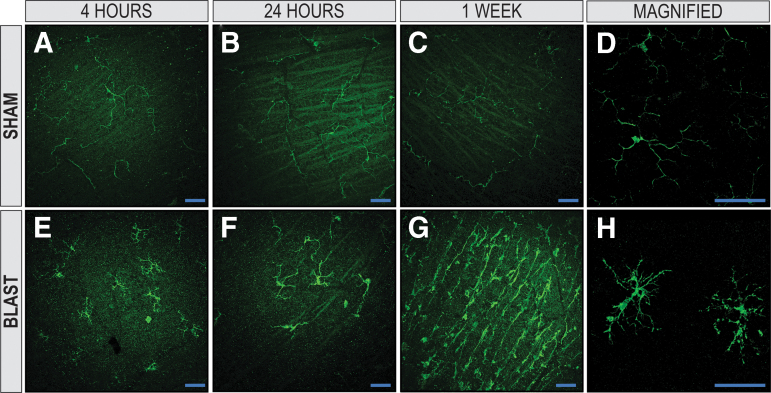
Microglia are activated in retinas of blast-injured animals. The IBA-1+ microglia in the retinal nerve-fiber layer (RNFL) and retinal ganglion-cell layer of retinal whole-mounts exposed to blast injury. Retinas from sham animals had a ramified morphology at 4 h, 24 h, and one week (**A**, **B**, and **C**, respectively). At 4 h, 24 h, and one week post-injury (**E**, **F**, and **G**, respectively), blast-injured retinas demonstrated hyper-ramified and bushy microglia, suggesting activation from injury. A two-dimensional *en face* view of a reconstructed z-stack acquired repeatedly in 0.5 μm confocal sections along the z-axis of retinal whole mounts demonstrates magnified representative images of ramified, resting sham and hyperramified, activated blast microglia shown in **D** and **H**, respectively (X63, original magnification). X25, original magnification for **A–C** and **E–G**; *en face* view with the RNFL facing up. Scale bar: 50 μm.

In sham mice, IBA-1 immunoreactivity showed ramified microglia with thin irregular processes, reflecting their resting state (Fig. 9A-D). When compared with sham, ipsilateral blast-injured retinas demonstrated microglia that were hyper-ramified and more bushy/hypertrophied with thicker processes and swollen cell bodies at all three time points (Fig. 9E-H), characteristic morphologic changes indicative of activation from injury.^50^

For the 24 h and one week time points, IBA-1 quantification (average of eight sample regions/retina; Fig. 2A) was performed to evaluate the percent area of fluorescence of IBA-1 positive microglia and the number of cell bodies present in the RNFL and RGC layers in sham and blast mice (representative images Fig. 2B, C, respectively). At 24 h, the percent area of fluorescence (Fig. 2D, bTBI = 3.15 ± 0.3044; sham = 1.93 ± 0.28; *p* = 0.0163) and the number of cell bodies (Fig. 2G, bTBI = 7.707 ± 0.5575; sham = 4.371 ± 0.6637; *p* = 0.0046) was significantly increased in the retinas of blast mice (Fig. 2F, I) when compared with sham (Fig. 2E, H; arrowheads indicate cell bodies), respectively.

This same finding was seen one week post-injury, with the percent area of fluorescence (Fig. 2J, bTBI = 5.065 ± 1.318; sham = 1.869 ± 0.04373; *p* = 0.0043) and the number of cell bodies (Fig. 2M, bTBI = 12.97 ± 2.797; sham = 5.019 ± 0.281; *p* = 0.0122) significantly increased in the retinas of blast mice (Fig. 2L, O) when compared with sham (Fig. 2K, N), respectively.

Astrocytes and Müller glia, the macroglia cells of the retina, carry out many homeostatic functions to maintain the retinal extracellular environment and health. Müller glia have processes that project radially throughout the entire span of the retina, aiding in the early detection of retinal stress, and provide limits at the outer and inner limiting membrane. In the healthy retina, GFAP is expressed abundantly in astrocytes in the RNFL and is localized to the end feet of Müller glia at the inner limiting membrane of the retina.^49^ In response to retinal injury or stress, however, GFAP immunoreactivity is increased in Müller glial processes and can extend throughout the retina,^51^ and has been seen in retinas of animal models after blast injury.^52^

We assessed the distribution of GFAP immunoreactivity in retinal cross sections to detect activation of Müller glial cells after blast exposure. At 24 h after injury, GFAP immunoreactivity was limited to astrocytes in the RNFL in ipsilateral retinas of both sham and blast-injured mice (Fig. 3A, B, respectively). At one week after blast exposure, the distribution of GFAP immunoreactivity remains in the RNFL of sham mice (Fig. 3C); however, it is consistently increased in bTBI retinas, spanning the retina radially from the RNFL to the outer nuclear layer (ONL; Fig. 3D). A two-dimensional side facing view of a reconstructed z-stack taken of whole-mounted retinas was rotated to demonstrate that GFAP immunoreactivity is limited to the RNFL of sham mice (Fig. 3E), while spanning the retina in blast mice (Fig. 3F) as a response to injury.

Quantitative plot profiles reflecting pixel intensity of GFAP staining in Fig. 3E and F demonstrate increased intensity in the blast retina (Fig. 3H) when compared with sham (Fig. 3G). Depth coded images of reconstructed z-stacks again demonstrate GFAP+ extension into deeper retinal layers of blast mice (Fig. 3J,L) when compared with sham (Fig. 3I,K). Quantification of the percent area fluorescence indicates that the blast group had higher GFAP immunoreactivity when compared with sham (*p* = 0.0014, *n* = 3; Fig. 3M).

### Anakinra prevents cellular activation after bTBI in the retina

**FIG. 10.**
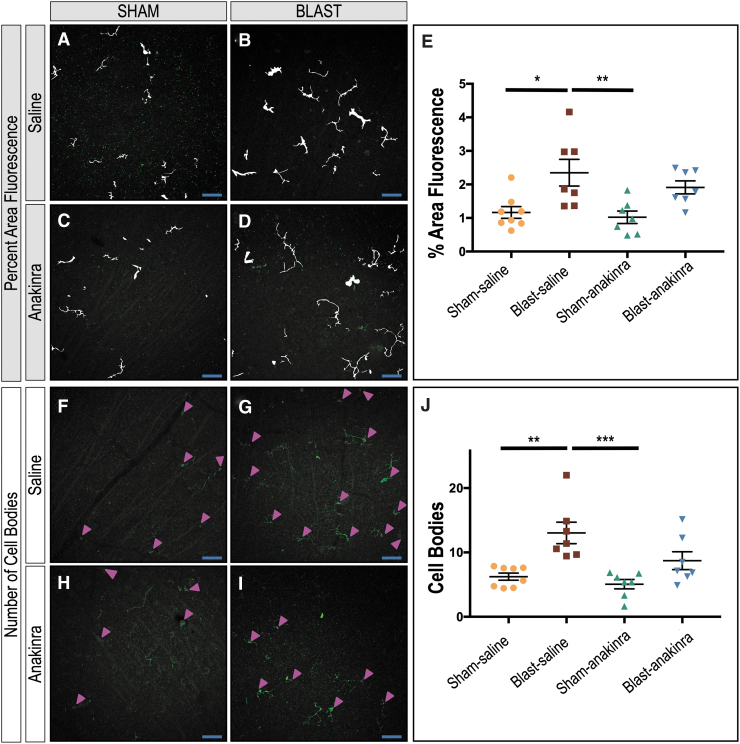
Anakinra treatment prevents microglial activation in the retina after blast. Mask overlays of the fluorescent area quantified (**A, B, C, D**) and arrowheads indicating cell bodies (**F, G, H, I**) for ipsilateral retinas of sham-saline, blast-saline, sham-anakinra, and blast-anakinra groups, respectively. Quantification shows a significant increase in total fluorescent area (**F**) and cell body counts (**J**) in the retinal nerve-fiber layer and retinal ganglion-cell layer of whole-mounted retinas from blast-saline mice when compared with both the sham-saline and anakinra-saline groups. No other significant differences were found. Significance determined comparing means of all groups using one-way analysis of variance with Dunnett post-test (*p < 0.05, **p < 0.01, ***p < 0.001). Data expressed as means ± standard error of the mean. Eight regions sampled per retina; *n* = 7–8 mice per group. X25, original magnification; scale bar: 50 μm.

To determine whether inflammatory blockade could decrease retinal damage after repetitive bTBI, we treated both sham and blast mice with either saline or anakinra (Fig. 6). We performed quantification on retinal whole mounts of the percent area of fluorescence (Fig. 10A–D) and number of cell bodies (Fig. 10F-I) of IBA-1 positive retinal microglia in the RNFL and RGC layers in response to blast injury four weeks after injury for our four conditions: sham-saline (SS), blast-saline (BS), sham-anakinra (SA), and blast-anakinra (BA), respectively.

When comparing the means of all groups, the BS group had significantly higher percent area of fluorescence than the SS and the SA groups (Fig. 10E, SS = 1.165 ± 0.1758; BS = 2.349 ± 0.3969; SA = 1.022 ± 0.1853; BA = 1.912 ± 0.1937; *p* = 0.0115 and *p* = 0.0057, respectively). No other significant relationships were found (SS vs. SA, *p* = 0.9763; SS vs. BA, *p* = 0.1676; BS vs. BA, *p* = 0.6250; SA vs. BA, *p* = 0.0896). The BS group also had a significantly increased number of cell bodies than the SS and the SA groups (Fig 10J, SS = 6.234 ± 0.5487; BS = 13.03 ± 1.669; SA = 5.063 ± 0.727; BA = 8.717 ± 1.385; *p* = 0.0013 and *p* = 0.0003, respectively), suggesting that anakinra treatment prevented microglia activation after blast injury. No other significant relationships were found (SS vs. SA, *p* = 0.8818; SS vs. BA, *p* = 0.4192; BS vs. BA, *p* = 0.0657; SA vs. BA, *p* = 0.1449).

We also assessed the effects of anakinra on Müller glia activation via the distribution of GFAP immunoreactivity in retinal cross sections four weeks after blast exposure in three separate mice per condition. Both the SS (Fig. 4A-C) and SA (Fig. 4D-F) groups demonstrated GFAP localization limited to the RNFL. Retinas from the BS mice exhibited increased GFAP immunoreactivity extending from the RNFL into deeper retinal layers, indicative of glial activation from retinal stress (Fig. 4G-I). The GFAP immunoreactivity was consistently visibly decreased in retinas from BA mice (Fig. 4J-L), however, suggesting that anakinra at least partially prevented Müller glia activation in blast-injured retinas.

Plot profiles of the pixel intensity of GFAP+ staining in Figure 4 demonstrate a decrease in signal intensity after anakinra treatment (Fig. 5). Quantification of the percent area fluorescence indicates that the BS group had higher GFAP immunoreactivity when compared with the SS, SA, and BA groups (*p* = 0.0089, *p* = 0.0065, and *p* = 0.0183, respectively; Fig. 5M). No other significant relationships were found (SS vs. SA, *p* = 0.9943; SS vs. BA, *p* = 0.9433; SA vs. BA, *p* = 0.8527).

### Anakinra prevents blast-induced RGC injury after bTBI

**FIG. 11.**
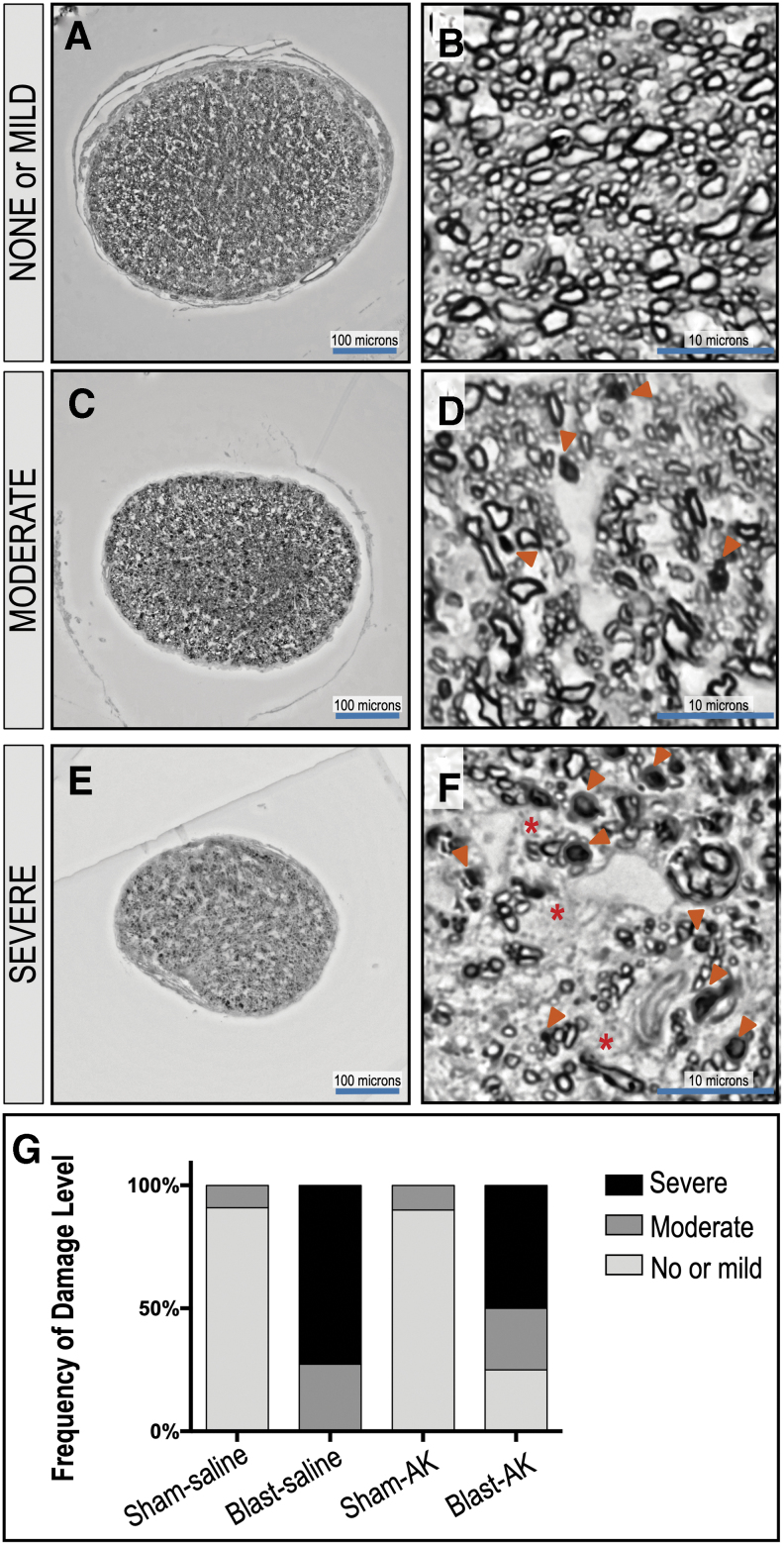
Anakinra treatment supports survival of axon bundles in the optic nerve after blast. The degree of neurodegeneration was assessed by analysis of the optic nerve axonal damage across sections of sham-saline, blast-saline, sham-anakinra, and blast-anakinra groups (*n* = 11, 11, 10, and 12, respectively). Representative low and high magnification image of ipsilateral optic nerve cross sections. Damage levels are based on grade. Grade 1: healthy-mild damage (**A, B**), Grade 2: moderate damage (**C, D**), Grade 3: severe damage (**E, F**). Arrowheads indicate paraphenylenediamine-positive and infilled dead/dying axons; asterisks indicate glial scar formation adjacent to glial cell nuclei. Frequency of damage level demonstrates that treatment with anakinra prevents damage of the optic nerve (**G**).

While anakinra decreased retinal glial activation after bTBI, we wanted to assess the changes in RGC structural and functional outcomes after blast with this treatment. RGC cell death can result in a loss of their axons compromising the optic nerve and resulting in visual field loss. We conducted histological analysis of optic nerves stained with PPD to highlight myelinated axons in sections of BS, SA, and BA groups (*n* = 11, 11, 10, and 12, respectively). The level of neurodegeneration was based on a grading system, with Grade 1 as healthy-mildly damaged (Fig. 11A, B), Grade 2 as moderate damage (Fig. 11C, D), and Grade 3 as severe damage (Fig. 11E, F) when evaluating PPD-positive and infilled damaged axons (arrowheads) and glial scar formation (asterisks) adjacent to glial cell nuclei.

The SS and SA optic nerves were most frequently healthy with Grade 1 damage (SS = Grade 1: 90.9%, Grade 2: 9.1%; SA = Grade 1: 90%, Grade 2: 10%), while the BS group was more frequently graded as more damaged (Grade 2: 27.27%, Grade 3: 72.72%). Treatment with anakinra, however, resulted in shifting the grading severity to lesser damage levels (Grade 1: 25%, Grade 2: 25%, Grade 3: 50%), suggesting that anakinra prevents some of the damage to the optic nerve that occurs from bTBI.

**FIG. 12.**
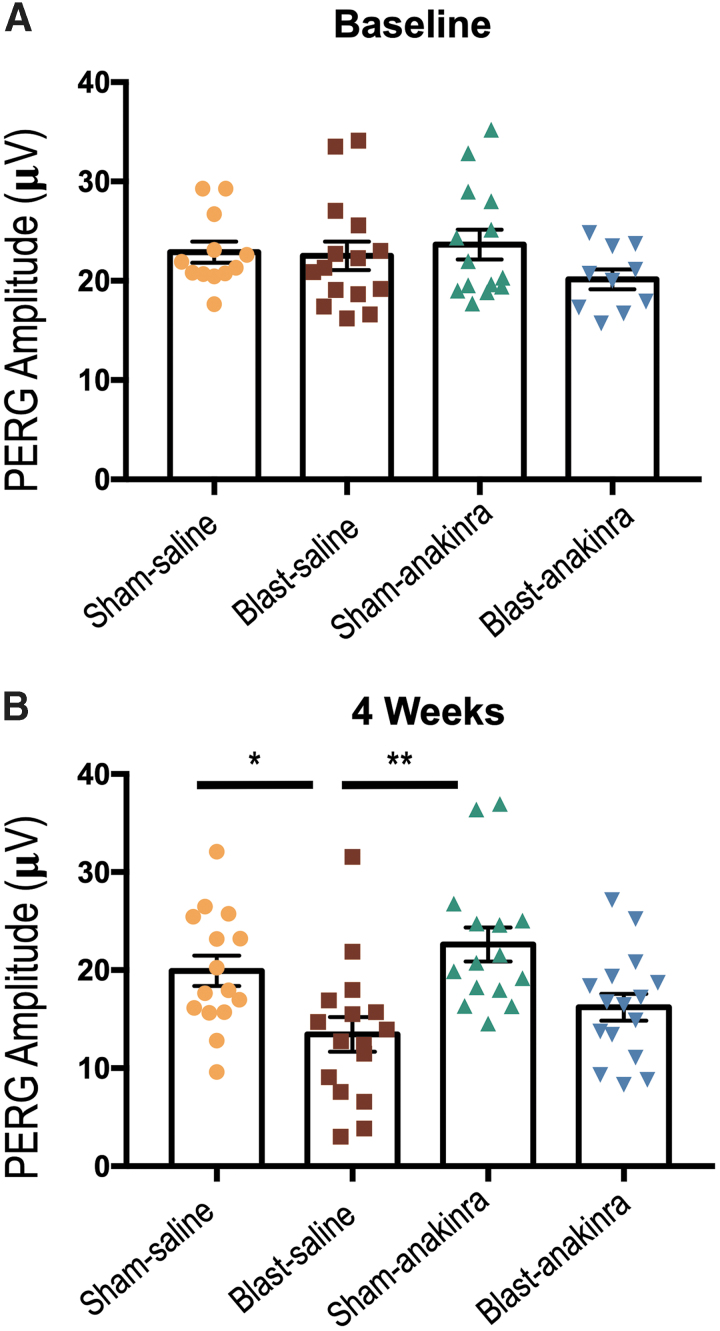
Blast-induced functional RGC damage post-repeated blast exposure is partially prevented by anakinra. There were no significant differences between groups at pre-blast baseline measurements (**A**). At four weeks post-injury, the pattern-evoked electroretinography (PERG) analysis revealed deficits in the blast-saline group, but not in the sham-anakinra or blast-anakinra groups (**B**), suggesting a partial rescue of RGC function. No other significant differences were found. All PERG recordings are of ipsilateral retinas and were measured in the neutral position. Significance was determined comparing means of all groups using Kruskal-Wallis test with the Dunn post-test (*p < 0.05, **p < 0.01). Data expressed as means ± SEM; *n* = 10–16.

The PERG amplitudes were utilized to measure the functional signaling capacity of RGCs. The pre-recorded baseline peak to trough amplitude for the ipsilateral eyes in the SS, BS, SA, and BA groups were 22.88 ± 1.055 μV, 22.51 ± 1.429 μV, 23.65 ± 1.506 μV, and 20.16 ± 1.007 μV, respectively, with no significant differences found when comparing all groups (Fig. 12A). At four weeks after injury, only the BS group had significantly decreased PERG peak to trough amplitudes when compared with the SS and SA groups (*p* = 0.0339 and *p* = 0.0012, respectively), suggesting that the blast group treated with anakinra had partially rescued RGC signaling (SS = 19.93 ± 1.546 μV; BS = 13.44 ± 1.768 μV; SA = 22.62 ± 1.74 μV; BA = 16.22 ± 1.37 μV; Fig. 12B). No other significant relationships were found (SS vs. SA, *p* = >0.9999; SS vs. BA, *p* = 0.8882; BS vs. BA, *p* = >0.9999; SA vs. BA, *p* = 0.1004).

**FIG. 13.**
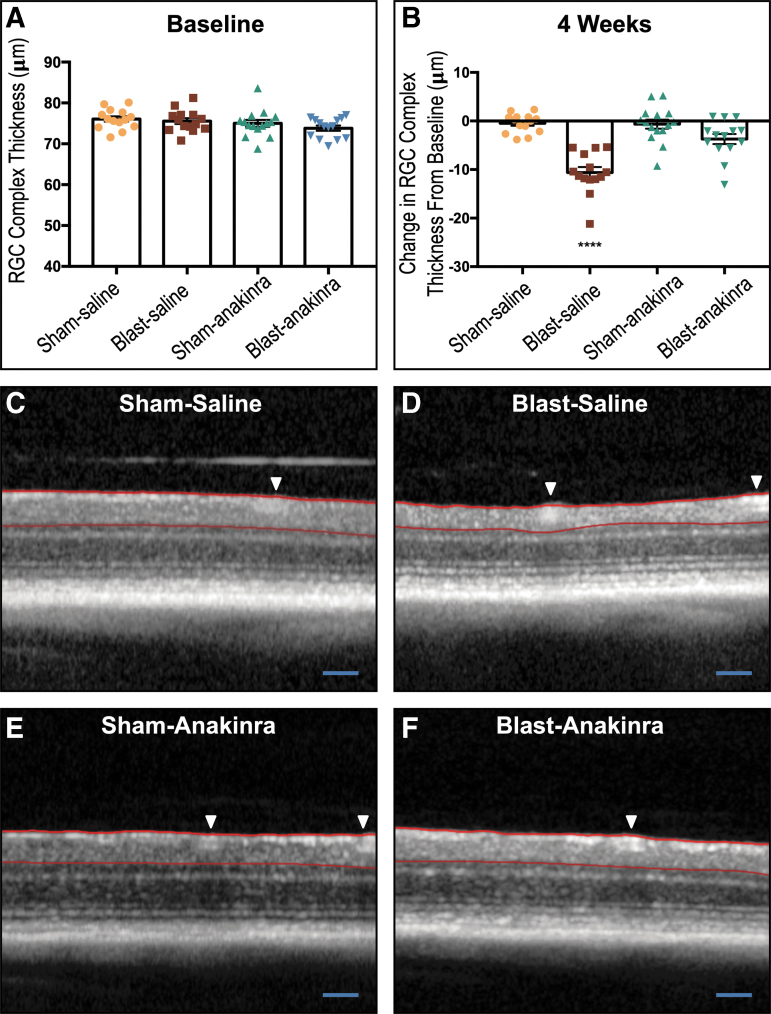
The retinal ganglion cell (RGC) complex loss from blast exposure is prevented partially by anakinra. The area between the red lines is the RGC complex thickness measured. No significant differences between groups were seen at pre-blast baseline (**A**). At four weeks post-injury, both sham groups and the blast-anakinra group had significantly less change in the RGC complex from baseline when compared with the blast-saline group, with no significant differences found between other groups (**B**). Representative optical coherence tomography images of sham-saline (**C**), blast-saline (**D**), sham-anakinra (**E**), and blast-anakinra (**F**). Arrows indicate blood vessels excluded from analysis. All data from ipsilateral retinas. One-way analysis of variance with a Dunnett post-test comparing all means (****p < 0.0001). Data expressed as means ± SEM; *n* = 14–16. Scale bar: 200 μm.

To evaluate the structural integrity of the retina, we used SD-OCT to measure the *in vivo* thickness of the RGC complex (RGC cell bodies, axons, and dendrites). The pre-blast baseline RGC complex thickness for the ipsilateral eyes in the SS, BS, SA, and BA groups were 76.07 ± 0.6511 μm, 75.58 ± 0.7159 μm, 75.01 ± 0.8511 μm, and 73.83 ± 0.6522 μm, respectively, with no significant differences found between groups (Fig. 13A). At four weeks after injury, the SS and SA and groups had average changes in RGC complex thickness of -0.4734 ± 0.532 μm and -0.6475 ± 0.9451 μm, respectively (representative images Fig. 13C and E).

By contrast, blast injury elicited a decrease in RGC complex thickness in the BS group (Fig. 13D) by -10.61 ± 1.131 μm that was significantly different from all other groups (*p* < 0.0001, Fig. 13B). After treatment with anakinra, the BA group exhibited preservation of the RGC complex, with a change from baseline of only -3.1714 ± 1.045 μm (Fig. 13F), suggesting that anakinra helped to maintain the structural integrity of the RGCs. No other significant relationships were found (SS vs. SA, *p* = 0.9992; SS vs. BA, *p* = 0.0868; SA vs. BA, *p* = 0.1057).

## Discussion

We have shown that repeated bTBI induces acute retinal inflammation, glial cell activation, and chronic neuronal dysfunction, which can be mitigated in part by IL-1 pathway blockade via anakinra. This current study validates our triple blast model because it induces similar cellular responses and damage as single bTBI models have demonstrated. In addition, the acute inflammation we see in the retina and brain is also seen in several other mouse models and human TBI,^15,19,27^ highlighting the pervasiveness of damaging secondary inflammation across models.

Other TBI studies targeting neuroinflammation and the IL-1 pathway within the brain have been promising, with decreased tissue damage and improved cognitive outcomes after injury.^25,26,28^ In another murine TBI model, the lateral fluid percussion injury model (FPI), our group has shown that reducing neuroinflammation via blockade of the IL-1 pathway does prevent cognitive damage within the brain.^27^ While current studies have not targeted the IL-1 pathway within the retina, other groups have shown that decreasing retinal inflammation via microglial polarization to the anti-inflammatory M2 state has protective effects after blast injury.^11–13^

Our group as well as several others has detected previously the presence of inflammatory cells in the retina after blast injury.^5,7,30,31^ In this study, we demonstrated the activation of microglia and Müller glia in response to blast injury. Because the retina is an immunoprivileged site, this hyperactivation of resident microglia (and possibly peripheral leukocytes because of blood-retinal-barrier disruption after blast)^53^ may contribute significantly to retinal tissue damage without an efficient way to regulate this immunological cascade. A study utilizing mice that lack a functional ocular immune privilege, allowing for increased retinal neuroinflammation, found injury after blast to be more severe than mice with an intact ocular immune privilege, suggesting again that inflammation contributes to retinal damage after TBI.^30^

Microglia can be activated by RGC damage, resulting in proliferation and migration to areas of damage within the retina.^48^ The morphological activation and increased number of microglia that we observed in the RNFL in our model suggest that microglia are responding to blast-induced RGC damage and that their activation is contributing further to neurodegeneration and cell death.

In the healthy retina, Müller glia prevent photoreceptor and neuronal cell death via the secretion of neurotrophic factors, growth factors, and cytokines.^49,54^ After blast injury, Müller glia demonstrated increased distribution of GFAP immunoreactivity, indicative of an injury response and retinal stress. Both activated microglia and Müller glial can contribute to retinal damage, because they are activated by and can continue to propagate damaging inflammatory signaling, acting as immunocompetent cells.

Modulation of glial activation through IL-1 blockade could be responsible for the improved visual outcomes in the setting of anakinra treatment after blast trauma. Histological analysis revealed that anakinra reduced the overall cellular activation of microglia and Müller glia four weeks after injury. This treatment maintained the signaling capability as well as the structural integrity of the RGCs and optic nerve. Preservation of RGC function and structure is necessary to maintain visual transduction from the retina to higher visual processing centers in the brain.

As with all animal models, there are variations and limitations of our bTBI model that should be noted. First, we utilize a head-only blast model in which the body is shielded from the blast wave via a PVC pipe to prevent damage to internal organs. While previous work has found that whole body exposure is an important contributor to blast TBI effects,^55^ visual and cognitive changes have been observed previously by our group in this model with only exposure of the head to the blast wave.^6,9,36,56,57^ These changes are similar to other investigations using head-only exposure.^5,14,52,58^ In addition, studies have shown similar positive phase pressure profiles in intact living mice and isolated heads of mice.^59^ This current study further validates that head-only blast exposure does yield visual deficits.

Second, we utilize a repetitive bTBI model as opposed to a singular injury model, because we are interested in studying the visual effects of mild, repetitive blast-induced TBI. Veterans are commonly exposed to multiple blast exposures during combat, further complicating their injuries.^37–39^ Our choice to utilize a repetitive bTBI model is reflective of human injury scenarios and is a paradigm regularly used by other groups studying blast-induced injury.^10,31,37^ We recently conducted a direct comparison of single to triple blast showing that both injury paradigms result in similar damage when assessing RGC layer thickness, RGC function, and total number of RGC loss.^60^

This current study demonstrated that damage to retinal ganglion cells can be detected after blast injury using non-invasive functional and structural tests, and that anti-IL-1 therapy through anakinra can confer partial protection to these cells. Pharmacological blockade of retinal inflammation could potentially be utilized to improve visual outcomes and quality of life in bTBI patients. The high dose of anakinra used in this study is not thought to cause any adverse effects in humans, because previous studies using high intravenous doses of this drug have not seen any changes in death or adverse outcomes in patients after stroke,^61^ subarachnoid hemorrhages,^62,63^ and even active bacterial sepsis.^64^ Future studies are needed, however, to determine the minimum effective dosage that should be administered to humans with bTBI.

While we evaluated the effectiveness of pre-treatment with anakinra in improving visual outcomes, this work does clearly implicate the IL-1 pathway as a contributing factor to retinal pathogenesis after bTBI. We identified an increase of retinal inflammatory cytokines at 4 h after injury; if treatment does indeed need to be initiated before this point, anakinra could still be effectively given by first responders, if not taken prophylactically by persons at high risk of blast exposure. Anakinra has an excellent safety profile even when used in individuals with active bacterial infections, making it an ideal cytokine blocker in this scenario because blast injuries can be accompanied by penetrating trauma depending on the device used and proximity to the blast epicenter. While the protective effects of anakinra were significant in this study, this drug alone does not completely prevent RGC damage.

Future studies will be aimed toward dissecting the IL-1 pathway to gain a greater understanding of involvement of each molecule in post-bTBI retinal inflammation, the timing of drug administration, and the possibility of combination therapy with other anti-inflammatory drugs used currently in humans (i.e., TNFα and IL-6) to manipulate them therapeutically after TBI and maximize visual protection.

## Supplementary Material

Supplemental data
