# Supplementary material for: Modulation of Post-Traumatic Immune Response Using the IL-1 Receptor Antagonist Anakinra for Improved Visual Outcomes
Source: J Neurotrauma. 2020 May 27;37(12):1463–80. doi: 10.1089/neu.2019.6725 (PMC7249480; doi:10.1089/neu.2019.6725)
Supplement: Supplemental data [file Supp_Table1.pdf]

Supplementary Data

Supplementary Table 1. Mice Utilized in Experiments*

Triple bTBI model validation

Cohort number	*Time point*	*Experiment*	n *per group*
1	4 h	RT-PCR: Retina	8–11
		RT-PCR: Brain	7–8
	24 h	RT-PCR: Retina	6
2	4 hr	Immunohistochemistry	
	24 hr	IBA-1 (whole mount)	5-6
	1 week	GFAP (cross section and whole mount)	3

Anakinra study

Cohort number	*Time point*	*Experiment*	n *per group*
3	Pre-blast and 5 week	PERG	10–16
		OCT	
		Optic nerve grading	10–12
		Immunohistochemistry	
		IBA-1 (whole mount)	7–8
		GFAP (cross section)	3

*All mice were aged 2–4 months and were C57BL/6J background.

RT-PCR, reverse transcription-polymerase chain reaction; IBA-1, ionized calcium binding adaptor molecule 1; GFAP, glial fibrillary acidic protein; PERG, pattern electroretinogram; OCT, optical coherence tomography,
